# Personalised External Aortic Root Support (PEARS) Compared with Alternatives for People with Life-Threatening Genetically Determined Aneurysms of the Aortic Root

**DOI:** 10.3390/diseases3010002

**Published:** 2015-01-15

**Authors:** Tom Treasure, John Pepper

**Affiliations:** 1 Clinical Operational Research Unit, Department of Mathematics, University College London, Gower Street, London WC1E 6BT, UK; 2 National Institute for Health Research (NIHR) Cardiovascular Biomedical Research Unit (BRU), Royal Brompton Hospital, Sydney Street, London SW3 6NP, UK

**Keywords:** Marfan syndrome, aortic root aneurysm, surgery

## Abstract

Personalised external aortic support was first proposed in 2000 by Tal Golesworthy, an engineer with familial Marfan syndrome and an aortic root aneurysm. After putting together a research and development team, and finding a surgeon to take on the challenge to join him in this innovative approach, he was central to the manufacture of the device, custom made for his own aorta. He was the patient for the ‘first in man’ operation in 2004. Ten years later he is well and 45 other people have had their own personalised device implanted. In this account, the stepwise record of proof of principle, comparative quantification of the surgical and perioperative requirements, 10 years of results, and development and research plans for the future are presented.

## 1. Introduction

Personalised External Aortic Root Support (PEARS) has undergone health technology appraisal by the National Institute for Health and Care Excellence [1]. After 10 years of clinical experience since the ‘first in man’ [2] a personalised support has now been implanted in 46 patients in three English hospitals and one Belgian hospital. Twenty of these patients were operated on more than five years ago and follow-up is now more than 200 patient years for the whole experience. The purpose of this paper is to summarise the evidence available to date. The PEARS study team believe that this surgery might now be made available to a wider group of patients but in a carefully controlled fashion as befits its novel and innovative status. Such an approach has been thoroughly described under the IDEAL recommendations [3,4,5,6,7,8].

### 1.1. Clinical Niche

This operation is intended to prevent on-going dilatation of an already morphologically abnormal aorta with the purpose of reducing the risk of death due to dissection in the aortic root. Most of the early patients have Marfan syndrome, but we see this operation as applicable to selected patients with root aneurysms due to Loeys-Dietz and Ehlers-Danlos syndrome and other genetically determined etiologies. It is the morphology of the aortic root aneurysm and the risk of dissection and rupture that are what we have to focus on in the present state of knowledge, not the genotype. PEARS might prove to be particularly applicable to patients early in their natural history.

Marfan syndrome is a rare disease estimated 20 years ago in a population in north east Scotland to have a minimal incidence of about 1 in 10,000 people, a minimal prevalence of 1 in 14,000 and about a quarter of cases were new mutations [9]. A recent report from Taiwan provides an estimate of 1 in 4286 which would potentially nearly double the number of operations [10].

People with Marfan syndrome are prone to aortic dissection and death predominately in their twenties and thirties. Before root replacement (effectively pre 1970) aortic dissection killed two thirds of all patients recognized as having Marfan syndrome [11,12,13]. Prophylactic root replacement potentially neutralizes that hazard and is the recommended management for patients who meet the widely accepted criteria defining them as being at high risk [14]. An estimate of the annual need for this surgery in UK is a mere 110 operations indicating the difficulty of accumulating a large series for observational studies or of finding candidates for a randomized trial (Table 1). Patients with other causes of genetically determined aortic root aneurysms which are considerably rarer than Marfan syndrome can also be considered as candidates for this surgery. To undertake a prospective comparative study, innovative study designs, of which we have experience in other fields of surgery, would be needed [15,16]. International collaboration would be required because of the relatively small numbers involved.

### 1.2. Existing Surgical Practice

Root replacement for congenitally determined aortic root aneurysms is available in two forms [17]. Each has advantages and limitations.

#### 1.2.1. Total Root Replacement (TRR)

First carried out in 1968 at Hammersmith Hospital in London, composite replacement of the aortic root and valve is generically known as the Bentall operation [18]. The operation as carried out now is very different to that first reported by Bentall in 1968. It has been refined during the subsequent 20 years [19] to become a highly reproducible operation, performed at low risk in expert hands. Its limitation is that a mechanical valve is usually used and there is an ensuing life-long risk of valve thrombosis. Anticoagulation is therefore mandatory and there is a consequent life-long risk of bleeding (Table 2). In an important meta-analysis by Benedetto the thromboembolism/bleeding event rate was 7% per decade. The overall rate of serious valve related events was 13% [20].

**Table 1:** Best estimates of the potential number of patients in the UK who may be candidates for aortic root surgery for congenitally determined aortic root aneurysms.

Statement
1. Marfan Syndrome has been estimated at 1:9800 [9] †
2. The UK population is 63.2 million
3. There are about 6500 people with Marfan syndrome in the UK
4. The average Marfan life expectancy is now >50 years and the Marfan Foundation (USA PPI view) say people with Marfan can live a normal life expectancy into their 70’s
5. The number of people becoming eligible for a root operation is estimated to be about 110 per year
6. About 85% of MFS patients might have an aortic intervention
7. About 110 aortic root operations are needed per year in the UK *

† These estimates are from 20 years ago. A recent report in the Mayo Clinic Proceedings [10] revises this estimate to 1:4286 which would potentially nearly double the number of operations. * It has proved difficult to disentangle the number of these operations currently done amongst the data for root replacement for other indications.

**Table 2:** Comparative demography and outcome data from meta-analysis compared with Personalised External Aortic Root Support (PEARS).

	TRR (972) *	VSRR (413) *	PEARS (30) **
**Patients**			
Mean age (years)	35	33	28 (IQR 20–44)
Mean pre-op Ao. root size (mm)	61	52	46 (IQR 43–48)
**Follow up events per decade**			
Re-intervention on aortic valve	3%	13%	None to date
Thrombo-embolic events	7%	3%	None to date
Composite valve events	13%	19%	None to date

* Data for TRR and VSRR from Benedetto *et al.* 2011 [20]; ** for PEARS from Treasure *et al.* 2014 [21].

In the early days in the 1970s, operative risk was considerable, as was reflected in the conservative thresholds for surgical intervention at the time. In 1981, when we as young surgeons were embarking on our careers, Johns Hopkins surgeons wrote *‘Until recently, surgical correction of Marfan defects of the aortic root has been undertaken with some hesitancy because of the high perioperative risk’* [22] but in that paper, after operating on 13 patients, they already saw the need to bring down the operative threshold to 5.5 cm. For the first 50 Bentall operations reported from Johns Hopkins in 1986 the smallest aortic size was 5.3 cm and the average size was greater than 7 cm [23]. Already the operative mortality in their hands was low; perioperative mortality approaching zero is now the expectation for composite root replacement.

#### 1.2.2. Valve Sparing Root Replacement (VSRR)

Forms of valve sparing root replacement were independently introduced by Yacoub in London [24,25,26] and David in Toronto [27,28,29]. The attraction of VSRR is that it spares the patient the need for life-long anticoagulation. Yacoub remodelled the aortic root with Dacron in a free-hand fashion. It is technically demanding and fell out of favour because of an unacceptable rate of progressive dilatation of the aortic annulus by the 10th postoperative year such that the valve became regurgitant. In David’s operation the aortic valve is enclosed within the tube graft to avoid this problem. It has had several iterations and has not proved consistently reproducible. Its limitation remains the technical challenge of valve conservation so the intended operation cannot always be completed and rescue valve replacement (TRR) is then done so the expected freedom from anticoagulation is not realised. In follow-up studies this is generally unreported so the intention-to-treat principle of reporting is defeated. The longer term failure rate of the conserved valves is about 13% per decade [20,30] (Table 2).

It is difficult to be certain of the mortality of valve sparing root replacement by intention-to-treat because it is in the nature of this surgery that in some cases it is ‘exploratory’ and only during the operation will it be clear whether or not the valve will be conserved. The only intention-to-treat analysis of which we are aware is the Aortic Valve Operative Outcomes in Marfan Patients Study (AVOOMP) report in which there was one death among 239 patients having valve sparing surgery (upper 95% confidence interval 2%) [31]. It is impressively low but we cannot be sure of the extent to which the intention was revised during the exploratory phase of surgery and whether this excellent low mortality is reproducible outside of the high quality collaborating centres [31].

There are many patients whose lives have been saved and survival extended by these operations but the perfect solution has not been reached. These are young patients, averaging around 30 at the time of surgery so nearly half are in their 20s. They may reasonably expect 50 years of life. During that time span the cumulative rates of valve related events, valve failure and reoperation have to be included ideally by intention-to-treat and should be considered in the advice and recommendations given to patients.

### 1.3. What Patients Value

#### 1.3.1. Avoidance of Anticoagulation Has a High Priority for Young Patients, Allowing Pregnancy, Active Sports and Normal Lifestyle

Avoidance of thromboembolic risk and anticoagulation was the motivation for surgeons to move from the reliable and highly reproducible operation that the modern Bentall had become during the 1990s to valve sparing root replacement which began to gather momentum in the 2000s following the reports from David [28]. PEARS shares the advantage of valve sparing root replacement in avoiding anticoagulation. In addition as a non-ablative operation it ‘burns no bridges’ and so there is an argument for offering it earlier in the evolution of aortic root aneurysm. It has the potential to save patients the anxiety of repetitive aortic root monitoring which may go on for years [32].

#### 1.3.2. Anticoagulation with Coumadin/Warfarin Is Regarded by Some as a Small Price to Pay

Modern methods of monitoring anticoagulation, including patient self-monitoring, have been associated with improved outcomes with respect to thromboembolism and bleeding [33,34]. However, a study from the department of Health Psychology at King’s College London (KCL) found that avoidance of anticoagulation was a strong and recurring theme for people with Marfan syndrome [32]. Daily medication and being monitored with blood tests made them feel they were in a ‘sick’ role; they wanted to be ‘normal’ people getting on with their normal lives.

Having the intervention sooner than might have been justifiable for root replacement was for them a benefit because of reduction in the anxiety associated with regular monitoring. There was a quantifiable reduction in anxiety (HADS: Hospital Anxiety and Depression Scale) after PEARS [32]. Reduction in hospital attendance and medication was for them a highly desirable outcome. The study is undergoing peer review for publication. It should be noted that the participants had all had PEARS surgery and were contributing to focus groups. To verify the finding in a wider patient group a further study is ongoing at KCL.

## 2. Experimental Section

### What External Support (PEARS) Entails

**Figure 1:**
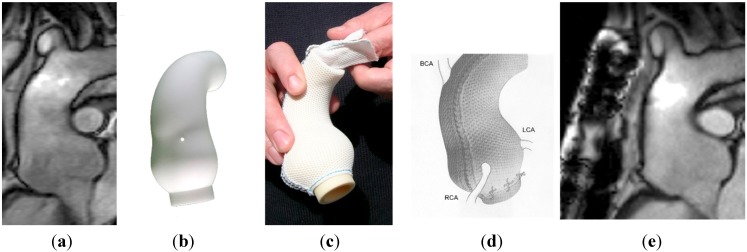
(**a**) The aorta before surgery. (**b**) The model on the computer screen after computer assisted design (CAD) modelling. (**c**) The soft macroporous mesh with 0.7 mm pore size mesh. (**d**) A depiction of the mesh surrounding the aorta and tethered to the ventricle with the coronary arteries emerging. (**e**) The MRI stable after 10 years.

The patient’s own aortic dimensions are used to manufacture a replica in plastic of the aorta on which a bespoke external support is made of a fabric mesh (Figure 1). This can be positioned around the aortic root and ascending aorta without cardiopulmonary bypass at a much shorter operation (Table 3) [35]. There is nevertheless a risk associated with surgery in this difficult anatomical territory. The operation entails dissection over the surface of the aortic root as far proximally as the aortic annulus. It is vital, therefore, to safely circumnavigate both coronary ostia and to be familiar with the three-dimensional anatomy of the aortic root. In one case a surgical injury to the left main coronary artery led to the only perioperative death (case 36 of 46 operations) a mortality rate on intention to treat of 2.2% (95% confidence intervals 0.5% to 11%) [21]. The patient had severe pectus excavatum and with hindsight the surgical teams think it was an instance where a brief period of cardiopulmonary bypass (<20 min as was used in the first patient and has been used occasionally in other patients) would have been warranted and might have saved a life.

The shape and intimate fit of the support make any movement unlikely. However, to ensure precise location the lower border of the support is folded on itself in a sutured seam. This is as inextensible as an annuloplasty ring and it is secured to the aortoventricular junction by six evenly spaced sutures.

**Table 3:** Comparison of peri-operative burdens of PEARS (first 30) with the other forms of root sparing root replacement.

	VSRR * (N = 239)	IQR	PEARS (N = 30)	IQR
Operation time minutes, median (IQR)	340	275–441	145	136–165
Bypass time minutes, median (IQR)	194	248–270	0	One patient
Myocardial ischaemia median (IQR)	156	117–221	0	0–0
Circulatory arrest	48 (20%)		0	0
Transfusion	Usual		1 (3%)	
Coagulation aid (FFP, platelets)	Common		1 (3%)	

* Data are from a prospective study of valve sparing surgery [36] compared with PEARS published data [21].

## 3. Results and Discussion

### 3.1. What External Support (PEARS) Offers

**Figure 2:**
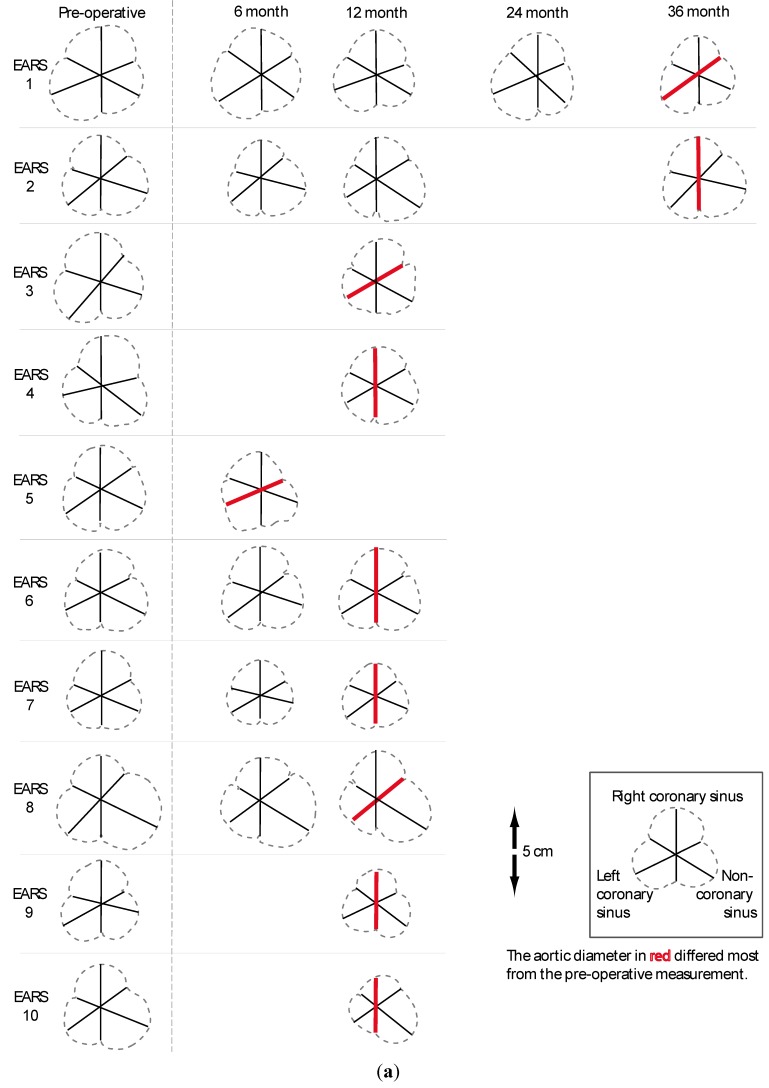

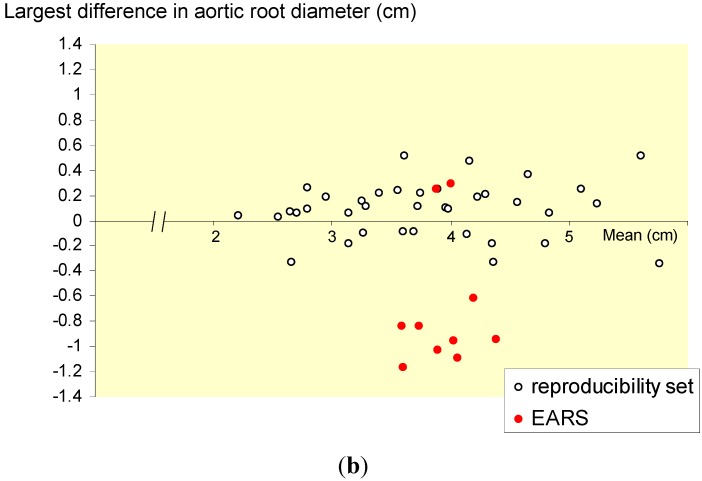
(**a**) Pictorial representation of cross sectional images at the level of leaflet coaption of all MRI studies in the first 10 patients. These were anonymised and presented in random sequence among duplicate copies of 37 unidentifiable Marfan studies. (**b**) A Bland and Altman plot. The average of the two readings is plotted on the Y axis and the difference between them on the X axis. Before and latest after PEARS images are shown in red. The 37 read/reread control images are in black. The greatest differences were used to maximise sensitivity to any change in size of the aorta in PEARS cases. It can be seen that variation of +/− 3 mm is common in a measurements made by an experience expert when blind to the clinical significance, indicating the possibility of bias in readings when the clinical context or research hypothesis is known to them as they make each measurement. Eight of 10 PEARS cases have a reduction in size after the aorta has been mesh supported.

PEARS has resulted in consistent technical efficacy, stabilising the aortic root and valve. This conclusion is based on multiple measurements of images before and after surgery in the first 10 recipients of external support, two at 36 months, seven at 12 months and one at six months. These 20 paired images were interspersed with duplicate images of 37 un-operated people with Marfan syndrome. In total 94 aortic root images were presented in random sequence to a radiologist with expertise in cardiac MRI [37] (Figure 2). Further radiographic comparison has been made in the first 24 patients operated on at Royal Brompton Hospital, 8 to 101 months (interquartile range 25.5–72 months) after surgery (Table 4). The morphology of the aorta supported by the mesh is quite unchanged over time.

Clinical outcomes at 1–10 years (average follow-up four years) are good. There have been no aortic or valve related events (Table 2). The PEARS operation is non-ablative, burns no bridges, and is a lesser biological insult than root replacement [38]. In two patients it has enabled a pregnancy [39].

**Figure 3:**
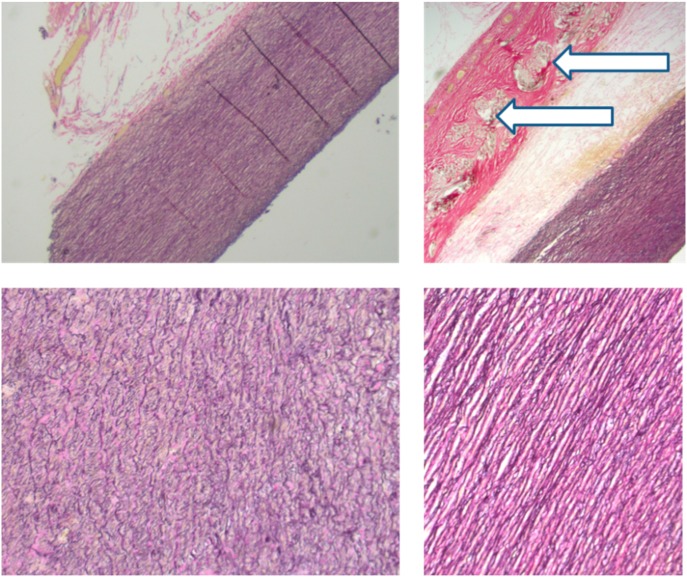
Histological appearances of the aorta post mortem 4.5 years after surgery. The images above: on the left the unsupported arch and on the right the supported root. (Magnification ×2.5) Below are the corresponding appearances of the media (×10). The arrows mark the mesh. Collagen fibres (stained red) pass through the interstices of the mesh which is incorporated in the adventitia. The histology of the arch (left) shows fragmentation while on the right the histological appearances are normal.

We have confirmed that the mesh is incorporated into the aortic adventitia (Figure 3). This is known from three sources. First, unknown to us at the time, a similar pliant macroporous material was used in an informal and incomplete wrapping of the aorta by a Californian team in the 1990s. Histology of samples of aorta obtained at re-operation showed the mesh to have been incorporated [40]. Second, material identical to that we have used clinically was used to sleeve the carotid artery of growing sheep. After an average of five months it was similarly incorporated with the connective tissues growing through the interstices of the mesh [41]. Third, one of the PEARS patients died in his bed at home 4.5 years after PEARS surgery [42]. There was no aortic dissection and the aortic valve was only minimally regurgitant as it had been throughout. His death was thought to be due to an arrhythmia associated Marfan cardiomyopathy, inherited from his mother. The unsupported aortic arch had the histological features of connective tissue disintegration, typical of Marfan syndrome while the supported portion of the aorta had normal histology [42] (Figure 3). This raises the intriguing possibility that the aorta, may have been able to heal when spared repetitive stress injury. What was confirmed in all three sources was that the mesh becomes incorporated in the adventitia forming a new composite vascular wall.

**Table 4:** Before and latest after PEARS aortic dimensions.

Mean of diameters N = 24	Before	Latest after
Aortic annulus (mm)	29	29
Sinus Valsalva largest diameter (mm)	45	44 *
Ascending aorta (mm)	32	33
Arch (mm)	24	24
Descending aorta (mm)	23	24

N = 24 available from 27 patients. * In eight of the first 10 patients there was a reduction in size in the aortic root because the support is positioned in the anaesthetised patient with the aorta under less tension. See also [37]. The descending aorta will increase in size with the passage of time (here an average of four years) in all patients and more so in Marfan syndrome. These differences are actually below reliable resolution on individual clinical measurement [37].

### 3.2. Cost Implications

Per operation PEARS is likely to save money because of the reduced procedural costs and avoidance of anticoagulation [43]. Fewer complications would also favour cost-effectiveness. However, intervening earlier would lead to more operations and inevitably increase the number needed to treat and thus the proportion of patients operated on who were never destined to dissect—but then earlier intervention would save lives now lost while waiting. Patients continue to die of dissection while knowing that the aorta is expanding. We have explored these issues elsewhere [43]. Further evaluation would be incorporated in a future prospective study.

## 4. Conclusions

For those whose valves cannot be spared, or who are operated in an emergency, PEARS is by definition, not an option. For elective prophylactic surgery we believe that this operation is sufficiently established in Britain [1] for a reasonable patient to expect to be informed. In Britain there is a duty to fully inform the patient. How this is interpreted will depend on all the circumstances but if a patient had an ablative operation, a decision to not inform the patient of this option might become questionable.

We proposed a prospective cohort study modelled on the Aortic Valve Operative Outcomes in Marfan Patients study [44] but with the inclusion of PEARS alongside TRR and VSRR. Such a study could theoretically provide decision making points amenable to randomization. The first is for patients who have not reached the criteria for root replacement. They could be offered random allocation between continued monitoring and PEARS, which can be justified in our opinion at lower aortic size. For patients who have reached the size criterion and have no more than trivial aortic valve regurgitation and are candidates for VSRR, the assignment to VSRR or PEARS could be made by randomization. This would require international collaboration to achieve the necessary numbers and wider acceptance of PEARS than is at present the case.

We know full well the difficulties of performing randomized studies in surgery [45] but they can and should be done if we are to have evidence rather than clinical judgment alone to help our patients towards the best course of action [46,47]. The proposed randomization points could be nested within a cohort study with a protocol based on prospective evaluation, with independent verification of outcomes, as was done in AVOOMP [31].
